# Genotype-specific agreement and accuracy of human papillomavirus DNA detection using Anyplex^TM^ II HPV28 and Xpert^TM^ HPV among women with HIV in Lusaka, Zambia: a diagnostic accuracy analysis

**DOI:** 10.1016/j.ijregi.2026.101027

**Published:** 2026-09-25

**Authors:** Nathalie Veronica Fernandez Villalobos, Misinzo Moono, John Andoh, Allison Glass, Partha Basu, Thamsanqa Madliwa, Mulindi Mwanahamuntu, Eliane Rohner, Nicola Low, Albert Manasyan, Katayoun Taghavi

**Affiliations:** 1 Institute of Social and Preventive Medicine, University of Bern, Bern, Switzerland; 2 Department of Reproductive, Maternal, Newborn, and Child Health, Centre for Infectious Disease Research in Zambia, Lusaka, Zambia; 3 Molecular Pathology Department, Lancet Laboratories, Johannesburg, South Africa; 4 Early Detection, Prevention and Infections Branch, International Agency for Research on Cancer, World Health Organization (IARC/WHO), Lyon, France; 5 Women and Newborn Hospital, University Teaching Hospital, Lusaka, Zambia; 6 Department of Pediatrics, University of Alabama at Birmingham, Birmingham, Alabama, USA

**Keywords:** HIV, HPV DNA test, Uterine cervical diseases, Women, Zambia

## Abstract

**Objectives:**

The prevalence of cervicovaginal high-risk human papillomavirus (hrHPV) is high among women with human immunodeficiency virus (WHIV). Many hrHPV tests exist, but genotype-specific comparisons among WHIV are limited. We compared hrHPV detection and diagnostic accuracy using Anyplex^TM^ II HPV 28 and Xpert^TM^ HPV among WHIV in Lusaka, Zambia.

**Methods:**

WHIV enrolled in a cervical screening test accuracy study (05/2019-03/2021) were invited for rescreening after 36 months. Trained nurses collected the cervical cytology and biopsy samples. Cytology samples were tested using Anyplex and Xpert. Women with paired and valid hrHPV results were included. We compared hrHPV and genotype-specific prevalence, overall agreement, Cohen kappa (κ), and relative sensitivity and specificity for the detection of high-grade squamous intraepithelial lesions (HSIL).

**Results:**

Among 230 women seen between 05/2022 and 06/2023, hrHPV prevalence was 24% (55/230) using Xpert and 36% (82/230) using Anyplex. Detection of HPV16 was higher with Anyplex (9%, 20/230) than with Xpert (6%, 13/230). Overall agreement was 85% (κ = 0.64; 95% confidence interval [CI] = 0.53-0.74). Genotype-specific agreement ranged from 88% (other hrHPV, κ = 0.66) to 96% (HPV16, κ = 0.70). For the detection of HSIL, the relative sensitivity of Anyplex compared with Xpert was 1.07 (95% CI = 0.94-1.21), and the relative specificity was 0.85 (95% CI = 0.79-0.91).

**Conclusion:**

Agreement between Anyplex and Xpert for hrHPV detection was high, but genotype-specific detection differed, with Anyplex detecting HPV16 more frequently than Xpert. While both assays had similar sensitivity, Xpert was more specific for HSIL. The choice of assay should balance the goals of maximizing HSIL detection with minimizing overtreatment.

## Highlights

• Agreement between Anyplex and Xpert was high for overall high-risk human papillomavirus detection.
• Anyplex detected more high-risk human papillomavirus than Xpert, with human papillomavirus 16 detected almost twice as often.
• Sensitivity was similar between both, but Xpert was more specific for detecting high-grade squamous intraepithelial lesions.

## Introduction

Reliable and accurate human papillomavirus (HPV) DNA testing, including genotyping for risk stratification, is essential to guide cervical screening and precancer treatment decisions among women with human immunodeficiency virus (WHIV), who are at high risk of cervical cancer [1]. Seventeen HPV genotypes are considered causal for cervical cancer [2,3]. Among these, HPV16 accounts for the highest global attributable fraction (61.7%), followed by HPV18 (15.3%) [2]. A better understanding of genotype-specific cancer risk has increased the use of HPV genotyping to inform risk-based screening and improve specificity.

The World Health Organization (WHO) recommends HPV detection as a primary cervical screening test [3]. WHIV have a higher risk of high-risk HPV (hrHPV) infection and faster progression to cervical cancer than women without HIV infection [4,5]. Multiple HPV infections are also about five times more common among WHIV [[6], [7], [8], [9], [10]]. Detection of transient infections reduces the specificity of HPV testing for detecting high-grade squamous intraepithelial lesions (HSIL) [[6], [7], [8], [9], [10]]. The WHO recommends HPV testing with triage among WHIV to guide treatment decisions and reduce harms such as overtreatment, anxiety, and adverse pregnancy outcomes [3,11].

More than 250 commercial HPV DNA tests are available, but few have been clinically validated or prequalified for use in cervical screening [12]. A 2020 systematic review found that seven assays, including Anyplex™ (Seegene, Seoul, South Korea) and Xpert™ HPV (Cepheid, Sunnyvale, CA), fulfilled the Meijer criteria for validating clinical performance [13]. The WHO has prequalified eight assays across six platforms for *in vitro* diagnostic use [[14], [15], [16]]. Xpert™ HPV is WHO-prequalified, whereas Anyplex™ is not [14,15]. Few studies have investigated the agreement between different HPV DNA tests among WHIV [17]. We compared the overall and genotype-specific agreement and test accuracy of Anyplex^TM^ II HPV28 and Xpert^TM^ HPV for the detection of HSIL among WHIV in Zambia.

## Methods

This study is reported in accordance with the Standards for Reporting Diagnostic Accuracy (STARD) 2015 guidelines (Supplementary Table 1).

### Study population

We invited all 375 WHIV who participated in a cervical screening test accuracy study between 05/2019 and 03/2021 at Kanyama Hospital in Lusaka, Zambia [18,19], to return for a follow-up 36 months later. At baseline, we included women aged 18-65 years with confirmed HIV infection who had ever had sex and were presenting for cervical screening [19]. Women with a history of cervical cancer, total hysterectomy, or prior HPV vaccination were excluded. Although yearly screening is recommended for WHIV, only 29% (*n* = 108/375) reported ever being screened prior to enrollment [18].

### Procedures

Trained nurses performed speculum examinations and collected cervical samples with a cervix brush (Rovers, Netherlands) placed into PreservCyt (Hologic Inc, USA). They then conducted visual inspection with acetic acid and colposcopy using a handheld device (Gynius AB, Sweden). All women had two to four colposcopy-directed cervical biopsies taken; if a lesion was present, two biopsies were taken from the most severe areas. If no lesions were seen, biopsies were taken from a random position within the transformation zone in all four quadrants. Blood samples were tested for cluster of differentiation 4 (CD4) cell count (Pima CD4 Analyzer, Alere, USA) and HIV RNA viral load (Cobas HIV-1/2 Qual; Roche Molecular Systems, USA).

### HPV DNA testing

PreservCyt samples were tested for hrHPV DNA using Xpert^TM^ HPV (Cepheid, USA, catalog number: GXHPV-CE-10) and Anyplex^TM^ (Seegene, South Korea, catalog number: HP7S00X). The Xpert HPV assay is a qualitative, multiplex polymerase chain reaction (PCR)-based test that detects 14 hrHPV genotypes with partial genotyping: (i) HPV16, (ii) HPV18 and 45, and (iii) “other hrHPV” (HPV31, 33, 35, 39, 51, 52, 56, 58, 59, 66, or 68). The limit of detection (LoD) of the Xpert HPV assay ranges between 10 and 30 copies per PCR reaction when evaluated using purified hrHPV DNA plasmids. The assay was validated using specific HPV-positive cell lines: HPV16 (SiHa), HPV18 (HeLa S3), HPV45 (MS751), and HPV68 (ME180). Xpert was run on-site within 24 h of collection on the GeneXpert platform with automated sample extraction using 1 ml of PreservCyt solution.

The Anyplex II HPV28 assay is a PCR-based nucleic acid amplification test that utilizes Dual Priming Oligonucleotide (DPO™) technology, involving tagging oligonucleotide cleavage and extension (TOCE™), to provide individual results for 28 HPV genotypes. The LoD of Anyplex was 50 copies/reaction for plasmid DNA and 500 cells/ml for SiHa and HeLa cells. Anyplex reported signal intensities based on cycle threshold (Ct) values: <31 cycles (+++ strong), 31-39 cycles (++ intermediate), and 40 cycles (+ weak) categorically using cyclic-CMTA (Catcher Melting Temperature Analysis). PreservCyt samples were tested by Anyplex within 2 weeks of collection at a South African laboratory. Both assays were performed according to the manufacturer’s instructions.

### Reference standard

Biopsy specimens were reviewed by expert pathologists at a South African laboratory using the WHO Classification of Tumors of Female Reproductive Organs, with p16 immunostaining performed [19]. HSIL was defined as histological HSIL, which includes HSIL (CIN2- p16-positive), HSIL (CIN3), adenocarcinoma in situ, or invasive cancer [20]. Pathologists were blinded to participants’ HPV results.

### Definitions and statistical analysis

We included data from women who returned for the 36-month follow-up visit and had valid paired hrHPV results. We restricted this analysis to the 14 hrHPV genotypes included in both assays. For Xpert, we recorded the results as shown in the test result window (HPV 16, HPV 18-45, Other hrHPV). Anyplex II HPV28 results were grouped to match the Xpert categories, referred to as Anyplex_Xr (Xpert-restricted) hereafter. We used descriptive statistics to characterize the study population and estimated test-specific hrHPV proportions with 95% confidence intervals (CIs). We calculated the percentage agreement in hrHPV detection between assays, overall, and stratified by cervical disease (<HSIL vs HSIL). We calculated Cohen kappa (κ) to assess agreement beyond chance between tests for any hrHPV and by genotype category, overall, and stratified by cervical precancer grade. We interpreted κ as follows: ≤0, no agreement; 0.01-0.20, slight agreement; 0.21-0.40, fair agreement; 0.41-0.60, moderate agreement; 0.61-0.80, substantial agreement; or 0.81-1.00, almost perfect agreement. We estimated CIs for κ using nonparametric bootstrap resampling with 1000 iterations. We examined Anyplex signal intensity (weak, intermediate, and strong) for individual hrHPV genotypes, stratified by Xpert HPV test result (positive, negative). For any hrHPV, we considered the highest intensity when multiple HPV genotypes were recorded. Data visualization and statistical analysis were performed in R version 4.2.2 (Supplementary table 2). We calculated the sensitivity and specificity for detection of HSIL for each test and estimated the relative accuracy of Anyplex_Xr compared with the Xpert HPV test.

### Sensitivity analysis

We performed a *post hoc* sensitivity analysis to assess the impact of Anyplex signal intensity on test accuracy, redefining Anyplex_Xr positivity to include only samples with intermediate or strong signal intensity. Using this definition, we calculated overall agreement, κ, and relative accuracy.

## Results

### Study population and hrHPV proportions

Follow-up visits were conducted between 05/2022 and 06/2023. Of the 375 consecutively enrolled women in the baseline study, 247 participants returned (Supplementary Figure 1). Among these 247 women, seven had invalid hrHPV results on either test, three did not have valid biopsy results, and seven were analyzed using another assay. A total of 230 women (median age: 41 years; interquartile range [IQR] = 35-48) were included in the analysis. Most participants had well-controlled HIV disease with an undetectable HIV RNA viral load at follow-up (Table 1). Almost a quarter (24%, *n* = 55/230) of the women who attended the 36-month follow-up had received cervical precancer treatment at the baseline visit.

**Table 1:** Characteristics of participants

Participant characteristics	Overall, n (%)
Number of participants^a^	230
Age, median (IQR)^a^	41 (35 – 48)
Age at sexual debut, median (IQR)	17 (16 – 19)
Marital status	
Single	7 (3%)
In a relationship/married	160 (70%)
Separated/divorced	35 (15%)
Widowed	28 (12%)
Contraception use	
Yes	89 (39%)
No	141 (61%)
Gravidity, median (IQR)	4 (3 – 5)
Parity, median (IQR)	4 (2 – 5)
Menopause	
Yes	36 (16%)
No	194 (84%)
HPV positive at baseline	
Yes	102 (44%)
No	128 (56%)
History of treatment for cervical precancer before enrolment	
Yes	5 (2%)
No	225 (98%)
Treatment for cervical precancer after baseline study visit	
Yes	55 (24%)
No	175 (76%)
CD4 cell count (cells/mm3), median (IQR)^a^	746 (586 – 953)
HIV RNA load (copies/mL)^a^	
Undetectable^b^	210 (91%)
Detectable	20 (9%)

a Based on evaluations performed during the current study visit

b Defined as <60 copies/mL

Of the 230 participants, 55 women (23.9%, 95% CI = 18.6-30.0%) tested positive for any of the 14 hrHPV genotypes using Xpert, and 82 women (35.7%, 95% CI = 29.5-42.2%) using Anyplex_Xr. Across different hrHPV categories, proportions were consistently higher using Anyplex_Xr than using Xpert. For example, HPV16 was detected in 5.7% (*n* = 13/230) of women using Xpert and 8.7% (*n* = 20/230) using Anyplex_Xr (Figure 1). Anyplex signal intensity was generally higher among Xpert-positive samples than among Xpert-negative samples (Figure 2). For most samples that tested positive for any hrHPV on both Xpert and Anyplex_Xr, Anyplex signal intensity was intermediate or strong (*n* = 42/51) (Figure 2 a). Samples that tested positive for HPV16 on both Xpert and Anyplex_Xr mostly had intermediate signal intensity on Anyplex (*n* = 9/15). For samples that tested negative on Xpert but positive on Anyplex_Xr, the signal intensity of Anyplex_Xr for the detection of any hrHPV was often weak (*n* = 20/31; Figure 2b). HPV16 was detected less frequently and showed a mix of intermediate (*n* = 1/5) and weak (*n* = 4/5) signal intensities.

**Figure 1:**
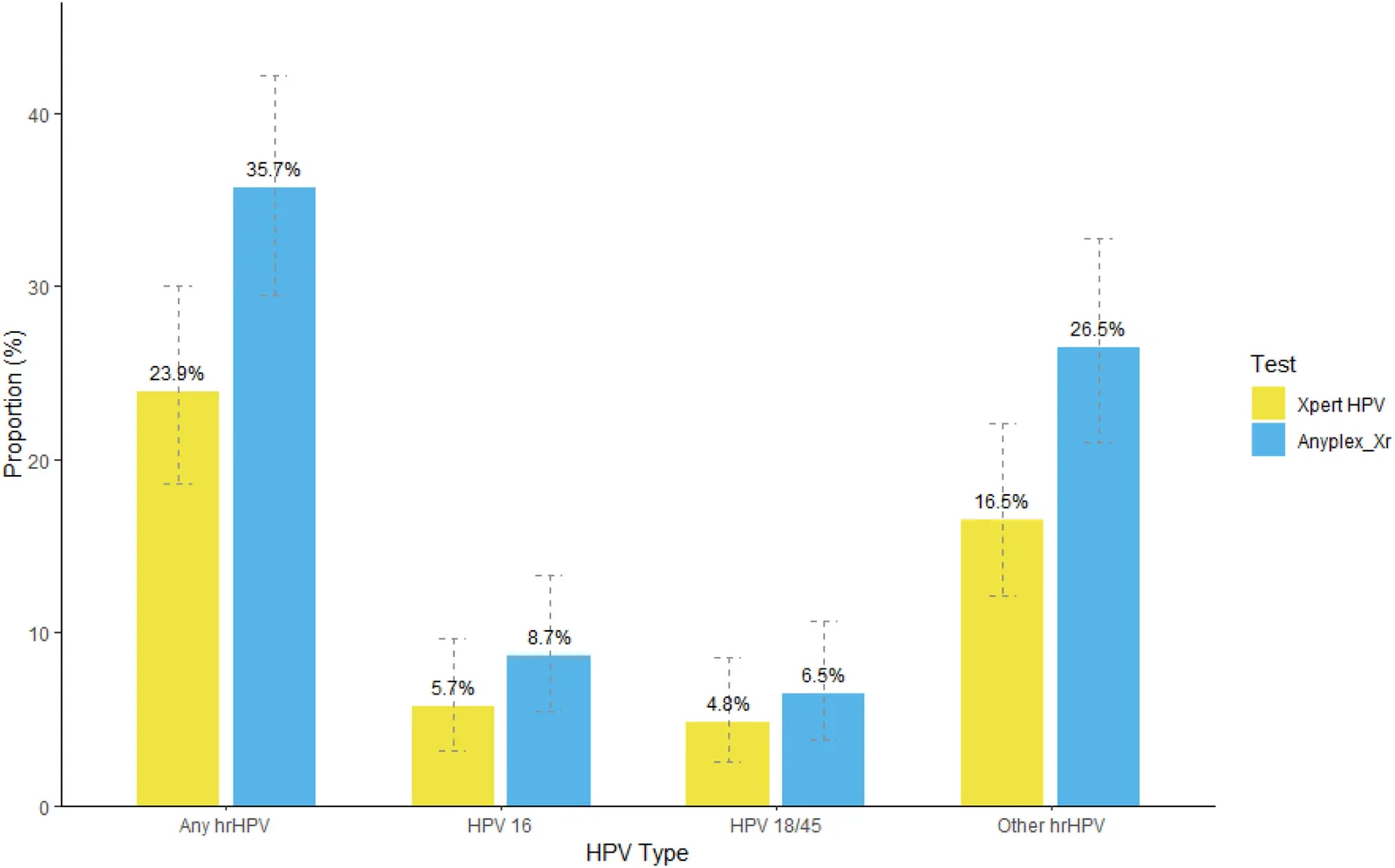
Proportion of positive samples for Anyplex_Xr^a^ and Xpert HPV, by hrHPV and subgroup-specific genotypes. Dashed lines represent 95% confidence intervals. ^a^Anyplex™ II HPV28 results were grouped to match the Xpert HPV genotype categories (*Anyplex_Xr, Xpert-restricted*). Abbreviations: HPV, human papillomavirus; hrHPV, high-risk human papillomavirus.

**Figure 2:**
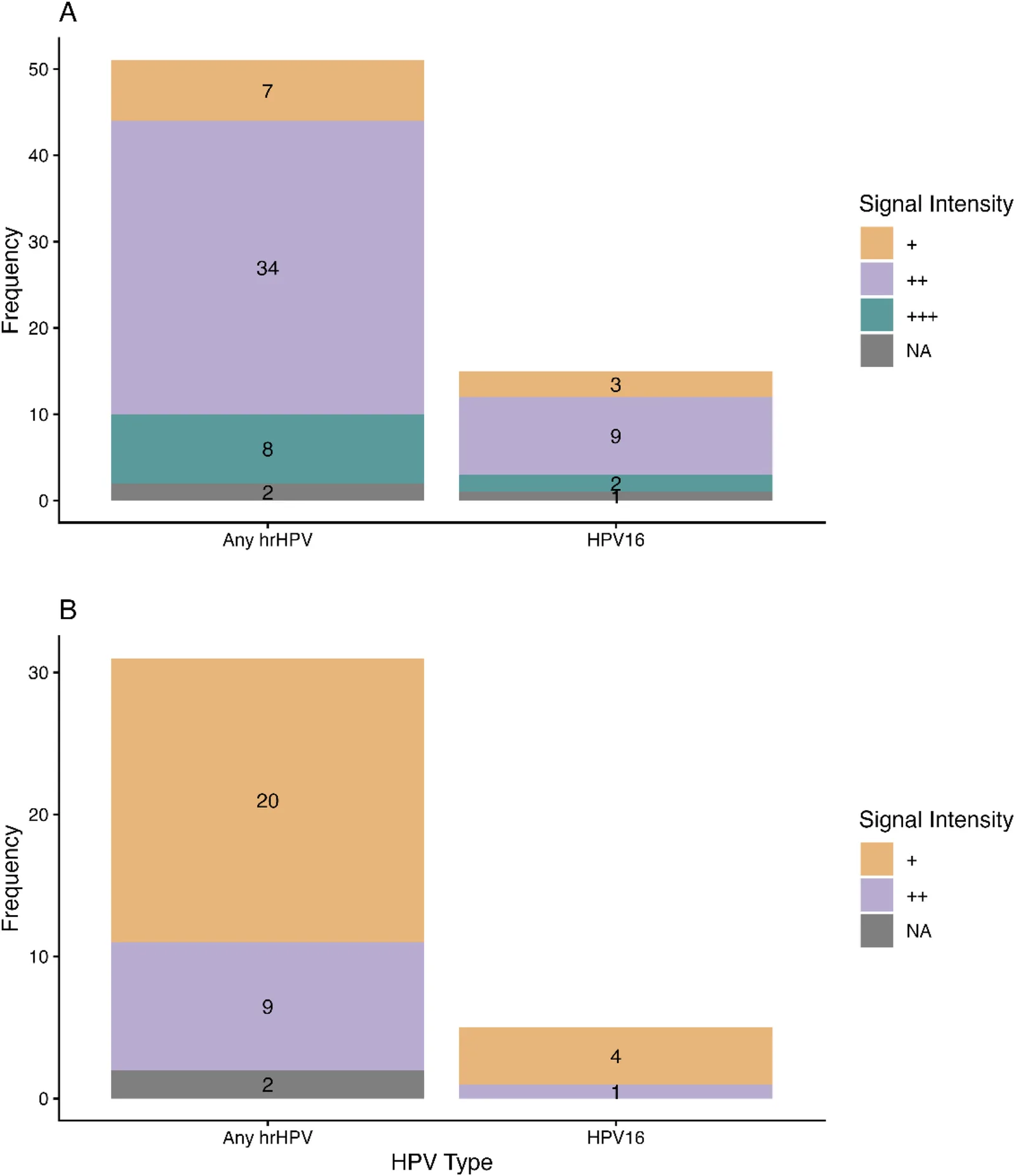
Distribution of Anyplex_Xr^a^ signal intensity for any hrHPV and HPV16, stratified by overall Xpert HPV test result: (a) Xpert-positive^b^ and (b) Xpert-negative. ^a^Anyplex™ II HPV28 results were grouped to match the Xpert HPV genotype categories (*Anyplex_Xr, Xpert-restricted*). ^b^ Xpert postitive results irrespective of genotype concordance with Anyplex_Xr. Abbreviations: hrHPV, high-risk human papillomavirus; HPV, human papillomavirus; NA, no signal intensity result.

### Agreement between tests

Overall agreement for detection of any hrHPV between Xpert and Anyplex_Xr was 85% (95% CI = 80-89%). Agreement was highest for HPV16 (96%, 95% = CI 93-98%) and HPV18/45 (95%, 95% CI = 91-97%), followed by other hrHPV (88%, 95% CI = 83-92%; Table 2). Agreement beyond chance was substantial for any hrHPV (κ = 0.64, 95% CI = 0.53-0.74), HPV16 (κ = 0.70, 95% CI = 0.50-0.87), and other hrHPV (κ = 0.66, 95% CI = 0.54-0.77), and moderate for HPV18/45 (κ = 0.51, 95% CI = 0.25-0.73). Overall agreement between tests was 95% (95% CI = 75-99%) among participants with HSIL and 84% (95% CI = 78-88%) among those with <HSIL (Table 3). Cohen kappa among women with HSIL was 0.83 (95% CI = 0.46-1.00), and 0.58 (95% CI = 0.44-0.70) among women with <HSIL. In the sensitivity analysis, considering only Anyplex_Xr results with intermediate to strong signal intensity as positive, overall agreement for any hrHPV was 90% (95% CI = 86-94%, κ = 0.73; Table 2).

**Table 2:** Agreement for HPV detection on cervical samples between Anyplex_Xr^a^ and Xpert HPV.

Anyplex_Xr	Xpert HPV result			Overall agreement (95% CI)	Cohen kappa (95% CI)
	Positive	Negative	Total		
**Any hrHPV**					
Positive	51	31	**82**	85% (80-89%)	0.64 (0.53-0.74)
Negative	4	144	**148**		
**Total**	**55**	**175**	**230**		
**HPV16**					
Positive	12	8	**20**	96% (93-98%)	0.70 (0.50-0.87)
Negative	1	209	**210**		
**Total**	**13**	**217**	**230**		
**HPV18/45**					
Positive	7	8	**15**	95% (91-97%)	0.51 (0.25-0.73)
Negative	4	211	**215**		
**Total**	**11**	**219**	**230**		
**Other hrHPV**					
Positive	36	25	**61**	88% (83-92%)	0.66 (0.54-0.77)
Negative	2	167	**169**		
**Total**	**38**	**192**	**230**		
***Sensitivity analysis of any hrHP****V*^b^					
Positive	42	9	**51**	90% (86-94%)	0.73 (0.61-0.83)
Negative	13	166	**179**		
**Total**	**55**	**175**	**230**		

Abbreviations: CI, confidence interval; HPV, human papillomavirus; hrHPV, high-risk human papillomavirus.

a Anyplex II HPV28 results were grouped to match the Xpert HPV genotype categories (*Anyplex_Xr, Xpert-restricted*).

b Anyplex_Xr positivity was defined as positive samples with a signal intensity of *intermediate (++)* or *strong (+++)*.

**Table 3:** Agreement for any hrHPV using Anyplex_Xr^a^ and Xpert HPV, by cervical disease status.

Anyplex_Xr	Xpert HPV result			Overall agreement (95% CI)	Cohen kappa (95% CI)
	Positive	Negative	Total		
**HSIL**					
Positive	15	1	**16**	95% (75-99%)	0.83 (0.46-1.00)
Negative	0	3	**3**		
**Total**	**15**	**4**	**19**		
**<HSIL**					
Positive	36	30	**66**	84% (78-88%)	0.58 (0.44-0.70)
Negative	4	141	**145**		
**Total**	**40**	**171**	**211**		

Abbreviations: CI, confidence interval; HPV, human papillomavirus; hrHPV, high-risk human papillomavirus; HSIL, high-grade squamous intraepithelial lesions.

a Anyplex™ II HPV28 results were grouped to match the Xpert HPV genotype categories (*Anyplex_Xr, Xpert-restricted*).

### Test accuracy

Xpert correctly identified 15 of 19 women with HSIL (Table 3) (clinical sensitivity 79%, 95% CI = 54-94%) (Table 4). Anyplex_Xr identified 16 of 19 women with HSIL, resulting in a clinical sensitivity of 84% (95% CI = 60-97%) and a relative sensitivity of 1.07 (95% CI = 0.94-1.21) compared with Xpert. Among 211 women with <HSIL, 171 tested negative for any hrHPV using Xpert (clinical specificity 81%, 95% CI = 75-86%). Using Anyplex_Xr, 145 women with <HSIL tested negative, resulting in a clinical specificity of 69% (95% CI = 62-75%) and a relative specificity of 0.85 (95% CI = 0.79-0.91) compared with Xpert. In the sensitivity analysis, Anyplex_Xr showed slightly lower sensitivity than Xpert (relative sensitivity 0.93; 95% CI = 0.82-1.07), but similar specificity (relative specificity 1.02; 95% CI = 0.97-1.07) for Anyplex_Xr-positive samples with an intermediate to strong signal intensity (Table 4).

**Table 4:** Relative sensitivity and specificity of testing positive for any HPV by Anyplex_Xr^a^ or Xpert HPV for the detection of cervical precancer (histological HSIL).

Test	Clinical sensitivity (95% CI)	Relative sensitivity^c^ (95% CI)	Clinical specificity (95% CI)	Relative specificity^c^ (95% CI)
**Xpert HPV**	0.79 (0.54-0.94)	1.07 (0.94-1.21)	0.81 (0.75-0.86)	0.85 (0.79-0.91)
**Anyplex_Xr**	0.84 (0.60-0.97)		0.69 (0.62-0.75)	
*Sensitivity analysis*^b^				
**Anyplex_Xr**	0.74 (0.49-0.91)	0.93 (0.82-1.07)	0.82 (0.77-0.87)	1.02 (0.97-1.07)

Abbreviations: CI, confidence interval; HPV, human papillomavirus; hrHPV, high-risk human papillomavirus; HSIL, high-grade squamous intraepithelial lesions.

a Anyplex™ II HPV28 results were grouped to match the Xpert HPV genotype categories (*Anyplex_Xr, Xpert-restricted*).

b Anyplex_Xr positivity was defined as positive samples with a signal intensity *of intermediate (++)* or *strong (+++).*

c *Relative sensitivity and specificity are for Anyplex_Xr compared with Xpert HPV.*

## Discussion

Among WHIV in Zambia, Xpert and Anyplex_Xr showed high overall agreement in detecting hrHPV genotypes. Anyplex_Xr detected hrHPV more often than Xpert, with HPV16 being detected more frequently. Sensitivity was similar between the assays, but specificity was higher for Xpert than for Anyplex_Xr. Most discordant results for individual hrHPV genotypes occurred when Anyplex_Xr’s signal intensity was low.

To our knowledge, this is the first study in sub-Saharan Africa directly comparing Anyplex and Xpert results among WHIV. A French study among WHIV reported hrHPV prevalence using Anyplex (77%) and Xpert assays (75%), but included both anal and cervical samples, complicating direct comparisons with our study [17]. Several sub-Saharan African studies evaluated Xpert and Anyplex among mixed populations including both WHIV and HIV-negative women, but provided limited evidence on agreement between the assays [[19], [20], [21]]. One study from Zimbabwe, which included 19% WHIV [21,22], reported an agreement of 77% (κ = 0.70), but this analysis was limited to Xpert-positive samples [21]. An Australian study used Anyplex and Xpert on self-collected and nurse-collected samples among women attending a colposcopy clinic to investigate within-assay agreement by sample collection modality [23]. Similar to our study, they reported a higher hrHPV prevalence in nurse-collected cervical samples using Anyplex (59%) than with Xpert (49%) [23]. Recent pooled test accuracy estimates from 28 studies published in 2022, which evaluated a range of HPV tests for the detection of CIN2+ among WHIV, found a slightly higher sensitivity (91.6%) and lower specificity (62.2%) than what we found for our assays. Of note, the pooled analysis included four studies using Xpert and none evaluating Anyplex [24].

A strength of our study is that both hrHPV assays were tested on the same nurse-collected specimens using standardized laboratory procedures. Furthermore, we reduced ascertainment bias for the detection of histological disease by obtaining 2-4 biopsies from each woman, including those with no visible lesion. Nevertheless, our study has limitations. Our relatively small total sample size, with only 19 HSIL diagnoses, limits the precision of estimates, particularly for sensitivity estimates. In this cross-sectional analysis, we could not examine the prognostic value of discordant or weak signal results for HSIL development. The Meijer criteria recognize that the absence of CIN2+ may be supported by longitudinal screening history, including repeated negative cytology or a previous negative HPV test. In the present study, disease status was assessed at the time of HPV testing through diagnostic evaluation, including 2-4 cervical biopsies in all women, irrespective of HPV test result. These approaches provide different information for determining disease status, and neither can establish the absence of CIN2+ with absolute certainty; this should be considered when interpreting estimates of clinical specificity. Finally, women in our study had been previously screened, and most had well-controlled HIV disease. Our results may therefore not be generalizable to all WHIV in sub-Saharan Africa.

For primary screening, maximizing sensitivity is favorable, whereas for triage, higher specificity may be preferable. We observed high overall agreement between Xpert and Anyplex_Xr results. Anyplex_Xr detected a higher proportion of samples with hrHPV, but this did not substantially increase the sensitivity of Anyplex_Xr for the detection of HSIL. Discordance was observed when Anyplex_Xr signal intensity was weak, which may be explained by differences in assay design. Anyplex provides genotype-specific results with signal intensities, whereas Xpert reports grouped hrHPV genotypes using channel-specific thresholds. The reported LoD varied between the assays and by HPV genotype, which may have contributed to differences in HPV detection. Differences in assay sensitivity across genotypes mean that infections with low viral loads, or HPV genotypes present at lower levels in mixed infections, may be detected by one assay but missed by the other. The assays also differ in sample preparation. Anyplex includes a separate DNA extraction and concentration step (Maxwell RSC Cell DNA Purification Kit), increasing DNA yield and potentially improving the detection of infections with low viral loads. Xpert tests the sample directly in the cartridge without a prior extraction step. Moreover, the amount of cervical cellular material collected and the corresponding HPV DNA concentration, may vary between samples, and the viral load in individual clinical samples is generally unknown [4,25]. Among WHIV, higher hrHPV prevalence, more frequent multi-type infections, and greater viral load variability may further contribute to discordance [4]. As our study population had previously undergone cervical screening and precancer treatment, few women had HSIL at this study visit. Previous precancer treatment may partly explain why HPV infections with low viral loads were common. When HPV viral loads are high, tests tend to agree more often [9,17,24]. In contrast, when viral loads are low, discordance increases because results often lie near or below the detection limit [9,17,24]. A 2024 systematic review found eight cross-sectional studies showing that low HPV viral load in cervical samples can be linked to less severe cervical disease on cytology [26]. The clinical significance of weak signal HPV positivity by Anyplex warrants further investigation. Prospective studies should investigate how signal intensity relates to HPV persistence and HSIL risk, particularly for HPV16.

Differences in sensitivity and specificity of HPV detection tests must be interpreted in the context of real-world screening programs. Where the priority is to detect and treat as many women with HSIL as possible, higher sensitivity of the primary test may be preferred. Conversely, in settings where treatment will follow a single positive result (“screen-and-treat”), higher specificity may reduce overtreatment. The number of HPV genotypes detected is also relevant. As HPV partial and extended genotyping are increasingly used for triage and management, agreement between assays needs to be evaluated. We found that Anyplex detected HPV16 more frequently than Xpert. In cervical screening programs where partial genotyping guides triage or immediate treatment, this would influence colposcopy burden and participant outcomes. Frameworks such as VALidation of HPV GENotyping Tests (VALGENT) provide benchmarks for HPV genotyping assays, but comparative data on assay performance among WHIV, particularly in sub-Saharan Africa, remain scarce [27,28]. Although HPV16, HPV18, HPV45, HPV33, HPV58, HPV31, and HPV52 account for most invasive cervical cancer globally, their combined attributable fraction is lowest in Africa. In contrast, HPV35 has a higher attributable fraction in Africa (3.6%) than in other regions (0.6-1.6%) [2,29]. Previous studies have also reported a strong association between HPV35 and cervical carcinogenesis among women of African ancestry [30], leading to calls for the development of HPV35-inclusive vaccines [29]. International clinical validation standards provide an important basis for establishing HPV assay performance. However, implementation should also consider the generalizability of test performance to the intended population, particularly where HIV prevalence, HPV genotype distribution, or disease prevalence differ from those represented in validation studies. Cost, turnaround time, usability, laboratory infrastructure, and program capacity are also important considerations [16,27,31].

In conclusion, among Zambian WHIV, Anyplex_Xr and Xpert HPV showed similar sensitivity for the detection of HSIL, while Xpert was more specific. Overall agreement was high, but assay-related differences in genotype-specific detection, especially for HPV16, could affect clinical management when genotyping guides triage or treatment. In settings with high HIV prevalence, assay selection should consider differences in HPV genotype detection as well as local resources and follow-up capacity. The decision should balance the goals of maximizing HSIL detection with minimizing overtreatment.

## Funding

Research was supported by the US National Institutes of Health’s National Institute of Allergy and Infectious Diseases; the Eunice Kennedy Shriver National Institute of Child Health and Human Development; the National Cancer Institute; the National Institute of Mental Health; the National Institute on Drug Abuse; the National Heart, Lung, and Blood Institute; the National Institute on Alcohol Abuse and Alcoholism; the National Institute of Diabetes and Digestive and Kidney Diseases; and the Fogarty International Center with grant number U01AI069924 and by the Swiss Cancer Research Foundation (KFS-4156-02-2017; KFS-5447-08-2021). K. Taghavi received funding from the Swiss National Science Foundation (P500 PM_210933). The content is solely the responsibility of the authors and does not necessarily represent the official views of the funders.

## Disclaimer

The content is solely the responsibility of the authors and does not necessarily represent the official views of the National Institutes of Health or the Swiss Cancer Research Foundation.

Where authors are identified as personnel of the International Agency for Research on Cancer/World Health Organization, the authors alone are responsible for the views expressed in this article and they do not necessarily represent the decisions, policy or views of the International Agency for Research on Cancer/World Health Organization.

## Data availability statement

Data are available on reasonable request to the corresponding author (katayoun.taghavi@unibe.ch) and with approval from all investigators.

## Ethics declaration

Ethical approval was obtained from the National Health Research Authority and the University of Zambia Biomedical Research Ethics Committee (014-09-18), the Zambia Medicines Regulatory Authority (DMS/7/9/22/CT/084), the IARC (IEC project number 18-15), and Swissethics (2018-01399 and 150/2014). All participants provided informed consent before their enrollment. Written and informed consent to take part in the study and to publish the results has been obtained from all participants or their legal representatives. The privacy rights of participants has been observed. This study includes human biological material and consent was obtained by donors or their next of kin or legal representatives for use in this study and for publication of the results. The samples used in this research were not sourced from executed prisoners or prisoners of conscience. This study was performed in compliance with relevant laws, regulations, and frameworks and guidelines where the research took place.

## CRediT authorship contribution statement

**Nathalie Veronica Fernandez Villalobos:** Writing – review & editing, Writing – original draft, Visualization, Methodology, Formal analysis, Data curation, Conceptualization. **Misinzo Moono:** Writing – review & editing, Validation, Supervision, Project administration, Investigation, Data curation. **John Andoh:** Writing – review & editing, Validation, Methodology, Formal analysis, Data curation. **Allison Glass:** Writing – review & editing, Validation, Investigation. **Partha Basu:** Writing – review & editing, Supervision, Investigation. **Thamsanqa Madliwa:** Writing – review & editing, Validation, Investigation. **Mulindi Mwanahamuntu:** Writing – review & editing, Supervision, Investigation. **Eliane Rohner:** Writing – review & editing, Supervision, Investigation, Funding acquisition, Conceptualization. **Nicola Low:** Writing – review & editing, Supervision, Investigation, Funding acquisition. **Albert Manasyan:** Writing – review & editing, Supervision, Resources, Project administration, Investigation, Funding acquisition. **Katayoun Taghavi:** Writing – review & editing, Writing – original draft, Resources, Project administration, Methodology, Investigation, Funding acquisition, Data curation, Conceptualization.

## Declaration of competing interest

The authors have no competing interest to declare.

## Appendix Supplementary materials

Image, application 1
