# Supplementary material for: Genotype-specific agreement and accuracy of human papillomavirus DNA detection using AnyplexTM II HPV28 and XpertTM HPV among women with HIV in Lusaka, Zambia: a diagnostic accuracy analysis
Source: IJID Reg. 2026 Sep 25;21:101027. doi: 10.1016/j.ijregi.2026.101027 (PMC13738138; doi:10.1016/j.ijregi.2026.101027)
Supplement: Supplementary file 1 [file mmc1.pdf]

Title

Genotype-specific agreement and accuracy of human papillomavirus DNA detection using Anyplex^{TM} II HPV28 and Xpert^{TM} HPV among women with HIV in Lusaka, Zambia: a diagnostic accuracy analysis

Short title

Comparison of high-risk HPV tests among women with HIV

Authors

Nathalie Veronica Fernandez Villalobos[1], Misinzo Moono[2], John Andoh[1] ,Allison Glass[3], Partha Basu[4], Thamsanqa Madliwa[3], Mulindi Mwanahamuntu[5] ,Eliane Rohner[1], Nicola Low[1], Albert Manasyan[2,6], Katayoun Taghavi[1, 4] *

Affiliations

1 *Institute of Social and Preventive Medicine, University of Bern, Bern, Switzerland*
2 *Department of Reproductive, Maternal, Newborn, and Child Health, Centre for Infectious Disease Research in Zambia, Lusaka, Zambia*
3 *Molecular Pathology Department, Lancet Laboratories, Johannesburg, South Africa*
4 *Early Detection, Prevention and Infections Branch, International Agency for Research on Cancer, World Health Organization (IARC/WHO), Lyon, France*
5 *Women and Newborn Hospital, University Teaching Hospital, Lusaka, Zambia*
6 *Department of Pediatrics, University of Alabama at Birmingham, Birmingham, Alabama, USA*

***Corresponding author**

Dr Katayoun Taghavi, Institute of Social and Preventive Medicine, University of Bern, Switzerland, katayoun.taghavi@unibe.ch

Supplementary Material

Supplementary Table 1. STARD 2015 checklist

Section/Topic	Item No.	STARD 2015 checklist item	Reported in manuscript?	Location in manuscript
TITLE OR ABSTRACT	1	Identification as a study of diagnostic accuracy using at least one measure of accuracy (e.g., sensitivity, specificity, predictive values, AUC)	Yes	Title/Abstract
	2	Structured summary of study design, methods, results, and conclusions (see STARD for Abstracts)	Yes	Abstract
INTRODUCTION	3	Scientific and clinical background, including intended use and clinical role of the index test	Yes	Introduction
	4	Study objectives and hypotheses	Yes	Introduction (end)
METHODS	5	Whether data collection was planned before index test and reference standard were performed (prospective) or after (retrospective)	Yes	Methods – Study population / Procedures
Participants	6	Eligibility criteria	Yes	Methods – Study population
	7	On what basis potentially eligible participants were identified (e.g., symptoms, previous tests, registry)	Yes	Methods – Study population
	8	Where and when potentially eligible participants were identified (setting, location and dates)	Yes	Methods – Study population; Results – Study population
	9	Whether participants formed a consecutive, random, or convenience series	Yes	Methods – Study population
Test methods	10a	Index test, in sufficient detail to allow replication	Yes	Methods – HPV DNA testing
	10b	Reference standard, in sufficient detail to allow replication	Yes	Methods – Reference standard
	11	Rationale for choosing the reference standard (if alternatives exist)	Yes	Methods – Reference standard
	12a	Definition of and rationale for test positivity cut-offs/result categories of the index test; distinguish pre-specified vs exploratory	Yes	Methods – HPV DNA testing; Definitions and statistical analysis; Sensitivity analysis
	12b	Definition of and rationale for test positivity cut-offs/result categories of the reference standard; distinguish pre-specified vs exploratory	Yes	Methods – Reference standard
	13a	Whether clinical information and reference standard results were available to performers/readers of the index test	Yes	Timing of results would not allow knowledge thereof

Section/Topic	Item No.	STARD 2015 checklist item	Reported in manuscript?	Location in manuscript
	13b	Whether clinical information and index test results were available to assessors of the reference standard	Yes	Methods – Reference standard
Analysis	14	Methods for estimating or comparing measures of diagnostic accuracy	Yes	Methods – Definitions and statistical analysis
	15	How indeterminate index test or reference standard results were handled	Yes	Results – exclusions described (invalid tests)
	16	How missing data on index test and reference standard were handled	Yes	Results – exclusions described
	17	Any analyses of variability in diagnostic accuracy, distinguishing pre-specified vs exploratory	Yes	Methods – stratified agreement; Sensitivity analysis (post-hoc)
	18	Intended sample size and how it was determined	No	Not reported<br>Observational cohort design
RESULTS	19	Flow of participants, using a diagram	Yes	Results text + referenced Supplementary figure 1
Participants	20	Baseline demographic and clinical characteristics of participants	Yes	Results; Table 1
	21a	Distribution of severity of disease in those with the target condition	Yes	Results (HSIL n=19); no CIN2 vs CIN3 breakdown
	21b	Distribution of alternative diagnoses in those without the target condition	No	None to report
	22	Time interval and any clinical interventions between index test and reference standard	Yes	Procedures -time windows for testing reported
Test results	23	Cross tabulation of index test results by reference standard results	Yes	Accuracy described; no explicit full 2×2 table vs histology shown
	24	Estimates of diagnostic accuracy and their precision (e.g., 95% CI)	Yes	Results; Table 4
	25	Any adverse events from performing the index test or the reference standard	No	None to report
DISCUSSION	26	Study limitations, including sources of potential bias, statistical uncertainty, and generalisability	Yes	Discussion – limitations paragraph
	27	Implications for practice, including intended use and clinical role of the index test	Yes	Discussion – implications and conclusion
OTHER INFORMATION	28	Registration number and name of registry	Yes	Parent study referenced
	29	Where the full study protocol can be accessed	Yes	Protocol cited
	30	Sources of funding and other support; role of funders	Yes	Funding statement

Supplementary Table 2. R-packages used for statistical analysis and data visualization

**Analysis**	**R add-on package**
Exact binomial 95% confidence intervals for the proportions were computed using the binom.prop.test() function.	Binom [1]
Wilson score confidence intervals (95%) were calculated for all agreement estimates using the BinomCI() function.	DescTools [2]
Cohen's kappa 95% confidence intervals were derived from the empirical percentile distribution of the bootstrap estimates using the boot() and boot.ci() functions.	Boot [3]
Sensitivity, specificity, positive predictive value (PPV), negative predictive value (NPV), and their respective 95% CIs were calculated using the epi.tests() function.	epiR [4]
Relative sensitivity and specificity were calculated using the tab.paired() function to structure the paired test data and the sesp.rel() function.	DTComPair [5]
Graphical display	gridExtra [6], ggplot2 [7], cowplot [8]

Supplementary Figure 1. Flow diagram of participant samples included in the study

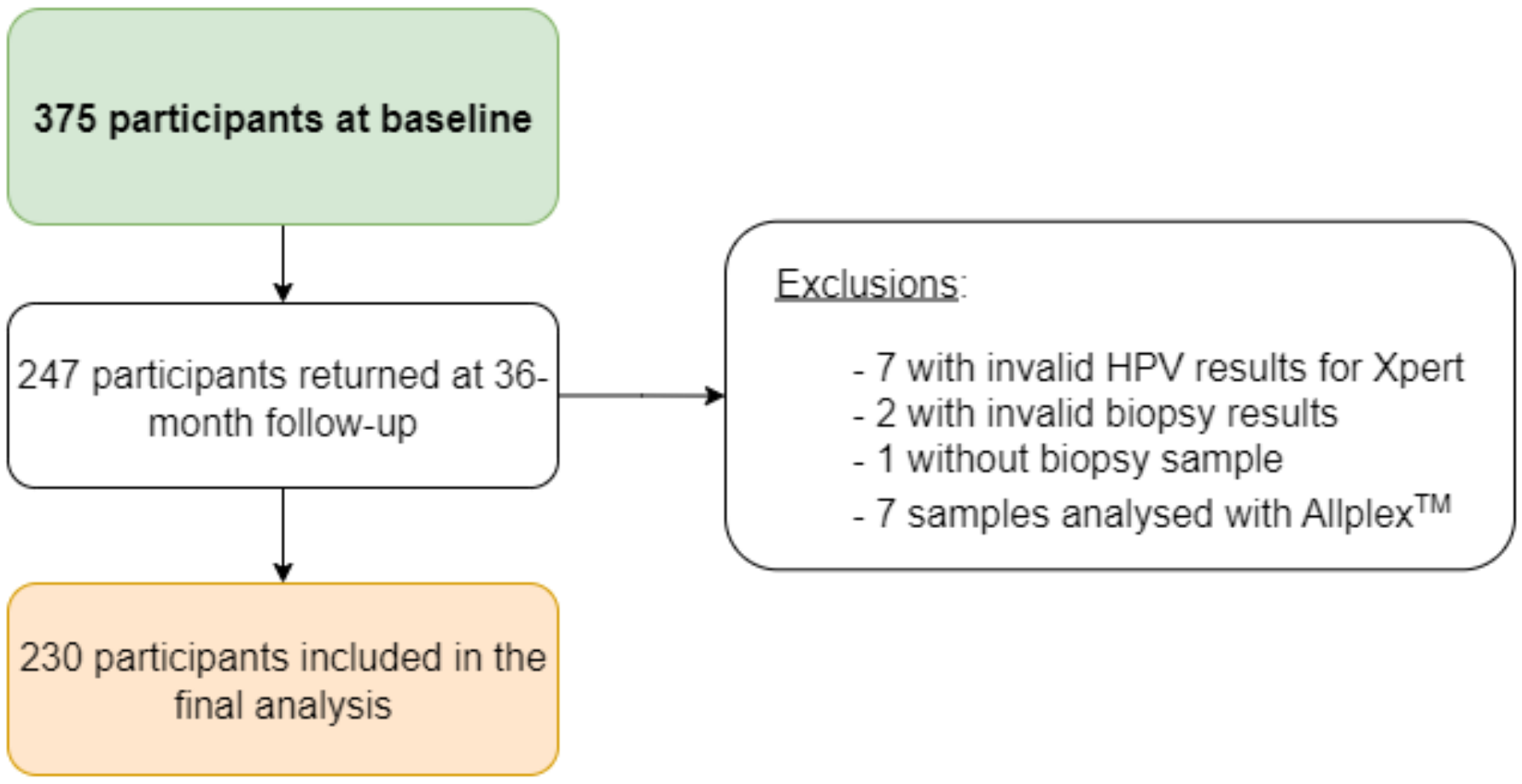

References

[1] Dorai-Raj S. Binomial Confidence Intervals for Several Parameterizations 2022.

[2] Signorell A. DescTools: Tools for Descriptive Statistics 2024.

[3] Canty A, Ripley B. boot: Bootstrap Functions (S-Plus) 2022.

[4] Stevenson M, Sergeant E. epiR: Tools for the Analysis of Epidemiological Data 2025.

[5] Stock C, Hielscher T, Discacciati A. DTComPair: comparison of binary diagnostic tests in a paired study design 2024.

[6] Auguie B. gridExtra: Miscellaneous Functions for "Grid" Graphics 2017.

[7] Wickham H. ggplot2: Elegant Graphics for Data Analysis. New York: Springer-Verlag New York; 2016.

[8] Wilke CO. cowplot: Streamlined Plot Theme and Plot Annotations for "ggplot2" 2020.
